# Algal Lipids as Modulators of Skin Disease: A Critical Review

**DOI:** 10.3390/metabo12020096

**Published:** 2022-01-20

**Authors:** Tiago Conde, Diana Lopes, Wojciech Łuczaj, Bruno Neves, Bruno Pinto, Tatiana Maurício, Pedro Domingues, Elżbieta Skrzydlewska, M. Rosário Domingues

**Affiliations:** 1 Centre for Environmental and Marine Studies, CESAM, Department of Chemistry, Santiago University Campus, University of Aveiro, 3810-193 Aveiro, Portugal; tiagoalexandreconde@ua.pt (T.C.); dianasalzedaslopes@ua.pt (D.L.); brunojpinto@ua.pt (B.P.); 2 Mass Spectrometry Centre, LAQV-REQUIMTE, Department of Chemistry, Santiago University Campus, University of Aveiro, 3810-193 Aveiro, Portugal; tatianascm97@ua.pt (T.M.); p.domingues@ua.pt (P.D.); 3 Department of Medical Sciences, Institute of Biomedicine–iBiMED, University of Aveiro, 3810-193 Aveiro, Portugal; bruno.neves@ua.pt; 4 Department of Analytical Chemistry, Medical University of Bialystok, Mickiewicza 2D, 15-222 Bialystok, Poland; wojciech.luczaj@umb.edu.pl (W.Ł.); elzbieta.skrzydlewska@umb.edu.pl (E.S.)

**Keywords:** skin diseases, inflammation, oxidative stress, lipidomics, bioactive lipids, anti-inflammatory, antioxidant, macroalgae, microalgae

## Abstract

The prevalence of inflammatory skin diseases continues to increase with a high incidence in children and adults. These diseases are triggered by environmental factors, such as UV radiation, certain chemical compounds, infectious agents, and in some cases, people with a genetic predisposition. The pathophysiology of inflammatory skin diseases such as psoriasis or atopic dermatitis, but also of skin cancers, is the result of the activation of inflammation-related metabolic pathways and the overproduction of pro-inflammatory cytokines observed in in vitro and in vivo studies. Inflammatory skin diseases are also associated with oxidative stress, overproduction of ROS, and impaired antioxidant defense, which affects the metabolism of immune cells and skin cells (keratinocytes and fibroblasts) in systemic and skin disorders. Lipids from algae have been scarcely applied to modulate skin diseases, but they are well known antioxidant and anti-inflammatory agents. They have shown scavenging activities and can modulate redox homeostasis enzymes. They can also downmodulate key inflammatory signaling pathways and transcription factors such as NF-κB, decreasing the expression of pro-inflammatory mediators. Thus, the exploitation of algae lipids as therapeutical agents for the treatment of inflammatory skin diseases is highly attractive, being critically reviewed in the present work.

## 1. Introduction

The skin is considered the largest organ in the human body with important roles for its homeostasis, such as protection against the harmful environment and dehydration [1]. The skin surface protects against pathogens, pollutants, and UV radiation [2,3]. These external stressors can pose a threat to the skin itself, promoting the formation of reactive oxygen species (ROS) that promote a pro-inflammatory response [4], associated with the pathophysiology of skin diseases, such as eczema, atopic dermatitis, psoriasis, vitiligo, and even skin aging or photoaging [5,6]. However, the pathology of skin diseases is not fully understood, and treatments are sometimes ineffective and inappropriate, with a low impact or with significant side effects [7]. Therefore, novel treatments are being sought out to fight the epidemic of skin inflammatory diseases. Synthetic drugs are not as effective as expected, and consumers are increasingly aware of using natural, organic, and environmentally friendly products. These new market trends and consumer preferences are stimulating the search for natural and sustainable ingredients (e.g., extracts of plants, microbes, or algae) to fight skin diseases [8,9]. The demand for natural molecules with beneficial properties for the skin includes the search for alternatives to synthetic molecules with fewer adverse side effects [10]. 

Algae (microalgae and macroalgae) are photosynthetic organisms considered natural reservoirs of compounds with bioactive properties, such as polysaccharides, vitamins, pigments, and polyunsaturated fatty acids (PUFA) [11]. These molecules are essential for the development of cosmeceuticals and are potential therapeutic agents with applications in skin diseases, including systemic or topical applications [12]. Algae biomass and algae extracts are already used in the treatment of skin diseases, for example, the use of macroalgae in thalassotherapy [13], or the use of algae oils rich in essential omega-6 and omega-3 PUFA (e.g., docosahexaenoic acid, DHA) in skin products [14]. Interest in algae-based products and natural ingredients has increased in the cosmetic and cosmeceutical industries in recent years [15]. Their main application has been as ingredients with hydrating, emollient, emulsifying, and whitening properties. Their use as anti-inflammatory and antioxidant products is also of great interest, but is less explored, and may be important for the management of skin diseases generally associated with periods of exacerbated inflammatory response and chronic inflammation [16].

Recent work has described the bioactive potential of algal lipids, namely their anti-inflammatory, antioxidant, antimicrobial, and antiproliferative properties [17,18]. They can be used for oral administration or incorporated into available products, or they can be used as extracts for topical application [15,19]. However, little is known about these properties of algal lipids and the detailed identification of bioactive lipids or the description of the relationship between their structure and their bioactivity is still missing. Nevertheless, the work described so far has shown that algal lipids are very promising and also constitute a sustainable alternative to replace ingredients of animal origin, in accordance with the United Nations Sustainable Goals and the European Green Deal [20,21]. This review describes the state of the art of the antioxidant and anti-inflammatory capacity of algal lipids and highlights their potential applications in the treatment of skin diseases.

## 2. Inflammatory Skin Diseases

Inflammatory skin diseases are the most common topical disorders, with increasing prevalence, posing both therapeutic and social challenges. In skin, the epidermis and dermis, in addition to the typical cells that build these layers, are populated by immune cells such as Langerhans cells, dendritic cells, macrophages, B and T lymphocytes, and NK cells, among others [22]. These immune cells in the presence of stressors or danger signals become activated and produce inflammatory mediators such as tumor necrosis factor alpha (TNFα), interferon-γ (IFNγ), interleukin (IL)-36, and IL-17, which induce a pro-inflammatory phenotype in keratinocytes, promote the recruitment of more leukocytes, and activate them, leading to a further increase in cytokine production [23]. In addition, some of these inflammatory mediators are involved in the metabolism of keratinocytes, in particular, their proliferation, differentiation, and apoptosis. Overactivation of the immune system can be caused by exogenous factors, such as UV radiation, some chemicals or microorganisms, accompanied by the increased production of sebum, for example, in cases of seborrheic dermatitis or acne [24]. However, in some cases, the source of the dysfunction of the immune system’s activity is not certain. These include autoimmune diseases such as psoriasis and atopic dermatitis (AD) [25]. These two diseases are the main inflammatory skin diseases, and their prevalence is still increasing. The incidence of AD and psoriasis is currently estimated at 20% in children and 10% in adults [25,26]. Currently, it is believed that the development of psoriasis is triggered by environmental factors, such as UV radiation, certain chemical compounds and infections in people with a genetic predisposition [27,28,29]. On the other hand, AD is caused by allergens which, in healthy people, do not activate the immune system. In psoriasis and AD, prolonged over-activation of immune cells leads to chronic inflammation that affects most skin cells but also affects the whole body. Inflammatory diseases are a growing problem among skin diseases as they not only cause cosmetic problems but also serious socio-psychological problems as well as various co-morbidities. In addition, they constitute a serious therapeutic problem since there is no permanent cure for these inflammatory diseases.

It is well known that the development of inflammatory skin diseases is accompanied by the recruitment and activation of neutrophils, which generate large amounts of ROS [30]. Nevertheless, under pro-inflammatory conditions, neutrophils generate ROS, even in the absence of pathogens, so these affect other tissues of the host, not only the skin. Among many types of ROS, the superoxide anion (O_2_^•−^), hydroxyl radical (OH^•^), and hydrogen peroxide (H_2_O_2_) are considered as the major species involved in development of inflammatory diseases [31]. The main source of O_2_^•−^ production in cells is mitochondria, where the transfer of electrons to molecular O_2_ is controlled. As a result of the manganese-superoxide dismutase (Mn-SOD) action, the formed O_2_^•−^ are metabolized into H_2_O_2_ [32]. However, OH^•^, generated from H_2_O_2_ in the presence of Fe^2+^ through the Fenton’s reaction, is considered as the most reactive ROS, leading to oxidative modifications of cellular components [33]. The accompanying weakened antioxidant defense leads to systemic oxidative stress and ROS not only exacerbate inflammation but are also involved in regulating immune cell function, for example, by promoting the differentiation of T lymphocytes into different subpopulations, depending on the level of ROS [34,35]. Other consequences of oxidative stress are oxidative modifications of cellular biomolecules. In such situation, ROS reacts with phospholipid PUFA, leading to lipid peroxidation and ultimately to the formation of reactive aldehydes such as 4-hydroxynonenal (4-HNE) and malondialdehyde (MDA) [36]. ROS may also indirectly modulate lipid metabolism, since lipid metabolizing enzymes are also over-activated under oxidative stress. Under oxidative stress conditions, structural modifications of proteins are generated. Thus, the activation of lipid-metabolizing enzymes is observed, leading to an increased generation of lipid mediators such as leukotrienes and prostaglandins, which are generally pro-inflammatory [37]. Moreover, the production of anti-inflammatory lipid mediators—endocannabinoids—is increased [38]. Additionally, modifications of antioxidant proteins [39], including those modulating the functioning of the transcription factors Nrf2 and NF-kB, responsible for the biosynthesis of antioxidant and pro-inflammatory proteins, leads to impairment of the cellular antioxidant defense and exacerbation of pathology in some inflammatory skin diseases, e.g., psoriasis [40]. Modification of the cytosolic Nrf2 inhibitor—Keap1 protein—prevents the formation of a complex with Nrf2 and promotes its transcriptional activity, leading to the biosynthesis of cytoprotective proteins, such as heme oxygenase 1 (HO-1), as observed in psoriasis [41,42]. In addition, in inflammatory diseases, ROS also activate other transcription factors, such as NFκB; and AP-1. NFκB can be activated by the p62-common activator of NFκB and Nrf2 and then regulates genes involved in inflammation, transformation, proliferation, and cell survival [43]. Thus, NF-κB via activation and increased pro-inflammatory cytokines production has been linked to various cellular processes in cancer [44]. These processes are also supported by the ROS-dependent activation of JNK, which leads to the synthesis of IL-1 and IL-6 in fibroblasts and the induction of collagenases [45]. Less is known about the role of AP-1, but current data suggest that it promotes cell growth and differentiation. Nevertheless, ROS can interact not only with transcription factors but also with other proteins, as well as with nucleic acids and especially lipids [46]. ROS react with phospholipids, in particular PUFA, which leads to lipid peroxidation with the generation of reactive aldehydes such as 4-hydroxynonenal (4-HNE) and malondialdehyde (MDA) [36].

As mentioned above, ROS and reactive aldehydes can impact different molecules and cellular functions. The fragmentation and disorganization of collagen fibers in the dermis not only leads to skin diseases such as erythema, oedema, and psoriasis but also the development of skin cancer [47]. Although the concept has not been fully elucidated, it has been proposed that the production of ROS is the result of three major mechanisms of carcinogenesis [48]. It has been found, among others, that the involvement of persistent oxidative stress in the development of melanoma and non-melanoma skin cancers results from the fact that ROS can activate oncogenes by the induction of proto-oncogenes and inactivation of certain protease inhibitors [49]. Regardless of the above mechanisms, ROS, by affecting the extracellular matrix of fibroblasts, lead to modifications of proteins that may also disrupt proliferation and induce mutations in skin cells. It is known that ROS can induce carcinogenesis by affecting the oncogenes BRAF, c-Myc, Ras, Rac-1, and the p53 suppressor gene. The BRAF and Rac-1 mutations are of particular importance for the development of melanoma, and the inhibition of Rac-1 may be mediated by ROS, which also direct the expression of HIF-1α in order to activate the Met proto-oncogene, which facilitates the angiogenesis, proliferation, and metastasis of melanoma [50]. Moreover, neoplastic transformation is mediated by signaling pathways regulated by ROS, especially PI3K/AKT and NF-κB, related to the regulation of inflammation and apoptosis [51]. On the other hand, ROS, participating in the induction of p53 mutations, also favor the development of other types of skin cancer, such as squamous cell carcinoma and basal cell carcinoma (SCC and BCC) [52].

In the course of various skin diseases, the effects of inflammatory processes are not limited to the changes in metabolism of skin cells but affect the whole body. Therefore, circulating leukocytes become overactivated and produce higher levels of cytokines. This leads to systemic inflammation and causes other co-morbidities. For example, psoriasis may exacerbate psoriatic arthritis, in which inflammation is observed in the joints [53,54].

It has been speculated that the main cause of the development of psoriasis is the increased proliferation of keratinocytes and the activation of immune cells due to pathological interactions between lymphocytes and epidermal keratinocytes induced by cytokines, such IFN-y and IL-22 [55]. In contrast, AD is the result of inflammation associated with abnormal allergic immune responses to allergens. T lymphocytes, as well as dendritic cells, are known to be key players in the pathogenesis of AD [56]. Acute AD is the result of inflammatory responses induced by Th2 and Th22, while Th1 responses are responsible for chronic AD [57]. The degranulation of mast cells in AD leads to the release of inflammatory mediators such as prostaglandins and leukotrienes. This situation leads to an exacerbation of the disease by recruiting eosinophils and lymphocytes to the dermis [58]. Despite differences in pathogenesis and an altered phospholipid profile, an increase in lipid peroxidation, as well as a decrease in the activity and level of enzymatic and non-enzymatic antioxidants in epidermal cells and plasma of psoriatic and AD patients, have been reported [59,60,61,62,63]. In addition, changes in the expression of transcription factors such as NF-κB, AP-1, or Nrf2 contributing to the alteration of the proteomic profile of the skin have also been observed in both diseases [64,65].

The significantly increased incidence of inflammatory skin diseases in recent years, including mortality associated with skin cancers and the lack of effective pharmacotherapy, is nowadays a global challenge, requiring further research to better understand the pathophysiology of these diseases and to develop new, more effective therapeutic solutions. Current treatments for skin diseases do not cure patients [29,66], and currently available treatments are generally cumbersome and complex to implement, and generate severe side effects. Thus, due to the close relationship between oxidative stress and the development of inflammatory skin diseases, future therapeutic approaches should rely on the use of preparations with antioxidant and anti-inflammatory properties, preferably of natural origin, that will reduce the side effects of the therapy.

## 3. Algae Lipids with Antioxidant Activity 

The antioxidant activity of algae occurring lipids has been studied, but is much less explored compared to well-known natural antioxidants such as pigments and phenolic compounds [67,68]. Published work on the antioxidant activity of lipid extracts of macroalgae and microalgae has been carried out *in chemico* and in vitro, focusing mainly on the evaluation of free radical scavenging activities against DPPH and ABTS radicals, the evaluation of the capability to decrease ROS levels, and modulation of specific enzymes and proteins involved in the regulation of oxidative stress (Table 1). 

*In chemico* assays have shown the antioxidant activity of lipid extracts of several species of macroalgae, such as *Ulva rigida*, *Codium tomentosum*, *Palmaria palmata*, *Gracilaria gracilis*, *Porphyra dioica*, and *Fucus vesiculosus*, among others, through the inhibition of ABTS and DPPH radicals [69]. In ABTS^●+^ assays, the IC50 values were found to range from 23.7 ± 0.6 μg. mL^−1^ for the lipid extract of *P. palmata* to 86.4 ± 3.4 μg. mL^−1^ for the lipid extract of *G. gracilis*. In the DPPH^●^ assays, the IC20 were in the range of 106.0 ± 5.6 6 μg. mL^−1^ for the lipid extract of *F. vesiculosus*, to 249.9 ± 66.7 μg. mL^−1^ for the lipid extract of *C. tomentosum*. Likewise, the antioxidant activity of lipid extracts of several species of microalgae was evaluated, such as using *Chlorella vulgaris*, *Chlorococcum amblystomatis*, *Scenedesmus obliquus*, *Tetraselmis chui*, *Phaeodactylum tricornutum*, *Spirulina* sp., and *Nannochloropsis oceanica* [70]. In ABTS^●+^ assays, the IC50 values were in a range of 29.4 ± 1.2 μg mL^−1^ for the lipid extract of *S. obliquus* to 101.9 ± 1.7 μg mL^−1^ for the lipid extract of *N. oceanica*. In DPPH^●^ assays, the achieved IC20 was ranging from 50.5 ± 12.3 μg. mL^−1^ for the lipid extract of *C. vulgaris* to 225.7 ± 6.9 μg. mL^−1^ for the lipid extract of *T. chui*. The values of IC reported in previous studies are of the same order of magnitude as those reported for phenolic compounds which are known for their distinct antioxidant action [71,72]. These studies, similar to most published work on the subject, did not include the identification or structural characterization of the bioactive lipids. They mainly focus on the FA composition and aim to establish a relationship between the FA profile and PUFA abundance with antioxidant activity [70]. Algae extracts are complex mixtures that comprise different classes of lipids, including neutral lipids, such as triacylglycerol (TAG), sterols, free FA; and polar lipids, such as phospholipids, glycolipids, and betaine lipids [73,74,75,76,77]. Recently, it has been suggested that polar lipids such as phospholipids, are better at delivering omega-3 PUFA [78]. Phospholipids increase the PUFA bioavailability due to their amphiphilic properties, which allow their efficient absorption in the intestines, incorporated into HDL-C and absorbed intact directly into the blood stream. Phospholipids integrated into plasma lipoproteins allow better PUFA delivery to tissues and organs than TAG. Furthermore, phospholipids can even cross the blood–brain barrier and deliver PUFA to the brain [79,80,81].

Different lipid fractions enriched from specific lipid classes were also assayed. For example, the neutral lipids fraction of macroalgae *Solieria chordalis* showed the best scavenging activity (DPPH^●^ assay, EC50 < 0.5 μg/mL^−1^) compared to glycolipids and phospholipids fractions [82]. Additionally, antioxidant activity has been reported in the C16-rich fatty acyl methyl esters FA from the green microalga *Scenedesmus intermedius*. This fraction inhibited 80% of DPPH radical at 40 μg. mL^−1^ and 70% OH radical scavenging activity at 40 μg. mL^−1^ [83].

In in vitro assays of the antioxidant activity of algae, the lipid extracts were tested, evaluating the capacity of the lipid extract to reduce the levels of ROS and modulate the enzymes involved in oxidative stress. The crude ethanolic extract of the macroalgae *Carpomitra costata* has been attributed as a photoprotective agent against ultraviolet B (UVB) in the human keratinocyte cell line HaCaT, promoting a reduction in the level of superoxide anions and hydroxyl radicals [84]. The ethyl acetate crude extracts from the microalga *Ettlia* sp. YC001 have shown high photoprotective effects against UVB radiation in normal human dermal fibroblasts (NHDF) by decreasing ROS levels in cells treated with hydrogen peroxide [85]. The crude fraction of the sulfoquinovosylacylglycerols (SQAG), rich in long-chain PUFA, from the red microalga *Porphyridium cruentum* showed the ability to decrease the levels of superoxide anions (IC50 as 29.5 μg. mL^−1^) in activated murine peritoneal mononuclear cells [86]. 

Lipid extracts have shown the capability to inhibit the expression of enzymes and proteins involved in the regulation of oxidative stress, such as metalloproteinases. Ethanolic extracts of *Arthrospira platensis* demonstrate protection against UVB in dermal fibroblasts [87] through the regulation of UVB-induced cytotoxicity and cell senescence by inhibiting the expression of thymine dimers, matrix metalloproteinase 1 (MMP1) and MMP3, important biomarkers of photoaging. Fucosterol from *Sargassum fusiforme* has shown potential for the prevention and treatment of skin ageing in human dermal fibroblasts. This was linked to the decrease in UVB-induced production of MMP1, IL-6, phosphorylation of c-Jun, and c-Fos and the increased expression of type I and transforming growth factor-1 (TGF-1) procollagen [88]. In addition, algal sterols down-regulate the expression of MMPs and type-I pro-collagen in UV-irradiated HaCaT cells by modulating mitogen-activated protein kinases (MAPKs) [89]

The pathophysiology of inflammatory skin diseases is associated with unregulated elevated levels of ROS and the activity of enzymes and proteins involved in the regulation of oxidative stress [30]. In cells, mitochondria metabolize oxygen, producing ROS. During the oxidative phosphorylation in mitochondria, oxygen is converted to O_2_^•−^, which can be transformed in H_2_O_2_ by superoxide dismutase, and then to water by glutathione peroxidase (GPX) or peroxiredoxin III (PRX III) radical [90]. Under normal conditions, the mitochondria ROS production is balanced by the production of a variety of antioxidants. However, oxidative stress occurs when there is an imbalance between ROS and antioxidants production. An imbalance in ROS production leads to redox signaling from cellular organelles, causing mitochondrial damage and dysfunction in several conditions [91]. However, the application of algae lipids to prevent mitochondrial dysfunction and modulate the oxidative status is little understood and requires in-depth study to understand the mechanisms underlying this potential antioxidant role. The use of crude extracts from algae may reduce ROS levels induced by UVB and impair the expression of MMPs and thymine dimers formation due to UVB exposure in skin cells [87]. These studies were performed using complex crude extracts rich in lipids and not with isolated lipids or fractions. This hinders the understanding of the mechanisms of action of algal lipids as antioxidants, and more work is needed to determine the potential protective role of algal lipids in skin diseases. A better understanding of this antioxidant action is needed, for example, there is a lack of knowledge about the impact of specific lipid classes or lipid molecules in the enzymes and proteins involved in the regulation of oxidative stress, such as metalloproteinases, HO-1, catalase, or superoxide dismutase. 

## 4. Algae Lipids with Anti-Inflammatory Activity 

The lipids of macro- and microalgae have been studied for their anti-inflammatory and immunomodulatory activity. Most of the studies tested crude lipid extracts or fractions of lipid classes of algae and were mainly performed *in chemico* and in vitro (Table 2). They mainly measured the impact of lipids on the levels of inflammatory effector molecules such as prostaglandins and nitric oxide (NO), cytokines such as TNF-α, IL-6, and IL-1β, and on the activation of inflammatory signaling pathways (NF-κB) or cyclooxygenase-2 (COX-2) activity. Few studies have evaluated in vivo models measuring the effect of lipids on skin cells, such as epidermal cells.

*In chemico* studies include the evaluation of the anti-inflammatory activity of extracts enriched in polar lipids of several species of macro- and microalgae, using COX-2 kit assay [69,92,93,94]. All the cited studies reported COX-2 inhibition, indicating the anti-inflammatory potential of algae extracts. The high inhibitory activity, in a few cases, was associated with the high content of omega-3 PUFA, as reported with extracts of the microalgae *Tetraselmis* sp. mutants IMP3 and CTP4, and *Skeletonema* sp. [95].

In vitro assays were performed using several immune cell lines, such as Raw 264.7, THP-1, or primary peripheral blood mononuclear cells (PBMC), and measured the impact on NO production after cell activation. The polar and non-polar lipids of the macroalga *Lobophora* sp. downregulated the expression of nitric oxide synthase (iNOS) and consequently decreased the production of NO [96]. Other studies have observed that the fractions enriched in phospholipids classes (PC and PG) isolated from the macroalga *P. palmata*, and the glycolipids classes (MGDG, DGDG, and SQDG) isolated from the macroalgae *P. palmata* and *Chondrus crispus* [97,98], and the microalgae *Nannochloropsis granulata* and *T. chui* [99,100] had a strong suppression of NO production in LPS-activated Raw 264.7 cells, this effect mainly being due to the decreased expression of iNOS. Similar trends were detected when these cells were exposed to other classes of lipids, such as free and ethyl esterified dihomo-γ-linolenic acid (DGLA, 20:3 omega-6) from a mutant strain (P127) of the microalga *Lobosphaera incisa* [101], DGTS from *N. granulata* [102], and MGMG from *Chlorella sorokiniana* [103]. 

Further in vitro studies evaluated the expression of COX-2 in Raw 264.7 and white blood cells using ethanolic extracts from the microalgae *C. vulgaris*, *Micractinium* sp., *P. tricornutum*, and *Chloromonas reticulata* [104,105,106,107]. The authors observed a decrease in the production of prostaglandin E2 (PGE2) as well as a downregulation of the expression of COX-2. Interestingly, one study reported increased base levels of PGE1, a prostaglandin associated with an anti-inflammatory response, in Raw 264.7 cells stimulated with free and ethyl-esterified DGLA [101]. 

Screening for inflammatory cytokine production is another parameter generally evaluated in in vitro studies screening the anti-inflammatory activity of algae lipids. Cytokines are soluble mediators responsible for regulating the inflammatory response through its boosting (pro-inflammatory) or attenuation (anti-inflammatory) [108]. The increased production of pro-inflammatory cytokines, such as TNF-α, IL-6, IL-1β, and IFN-γ, is also associated with inflammatory skin diseases [109]. The use of lipid extracts and lipid fractions isolated from algae promoted the down-regulation of these cytokines when activated by pro-inflammatory signaling in an immune cell model (THP-1) and skin cells (HaCaT) [110,111]. Most studies used complex crude extracts of microalgae (e.g., *Aurantiochytrium mangrovei, C. vulgaris, C. reticulata, Micratinium* sp., *Nitzschia palea, P. tricornutum, Spirulina maxima*, and *Tetraselmis suecica*) and observed the inhibition of the LPS-induced production of important pro-inflammatory cytokines, such as TNF-α, IL-6, and IL-1β [104,105,106,107,112,113,114,115,116]. Mosxou et al. reported that ethanolic extracts of the microalga *P. tricornutum* encapsulated in rice flour decreased the relative gene expression of iNOS, IL6, IL-1β, IL10, TNF-α, and NF-κB in NHDF stimulated by LPS [117]. Ethanolic extracts of the macroalga *Prasiola japonica* promoted the downregulation of mRNA expression of the inflammatory genes of IL-1β, IL-8, IL-6, TNF-α, and IFN-γ, as well as the suppression of the NF-κB pathway in HaCaT cells irradiated by UVB [111]. In addition, lipid extracts enriched in the glycolipids classes, such as SQDG, MGDG, and DGDG from the macroalgae *P. dioica*, *P. palmata*, and *C. crispus* and the microalga *Pavlova lutheri*, downregulated the pro-inflammatory production of IL-6, as well as the expression of Toll-like receptors (TLR) and their signaling pathways [110]. Phospholipids from algae have also shown the ability to inhibit the release of pro-inflammatory cytokines. The lysophospholipid LPC(16:0) from the microalga *Cylindrotheca closterium* decreased the LPS induction of TNF-α in THP-1 cells [118]. Another study showed that the lipid extract of the macroalga *Laminaria ochroleuca*, mainly composed of phosphatidylcholines (PC), decreased the release of cytokines IL-1α and IL-6 as well as prostaglandin PGE2 in an in vitro model of epidermal cells [119]. Interestingly, PC have previously been used as liposomes for the delivery of active molecules due to their ability to overcome the stratum corneum barrier [120], which presents a potential use for topical applications of formulations enriched with these polar lipids. Other lipids isolated from microalgae, such as oxylipins, ergosterol, and 7-dehydroporiferasterol, and DGLA (free and ethyl esterified), were responsible for the decrease in the production of LPS-activated pro-inflammatory cytokines [101,121,122]. Studies reporting anti-inflammatory activities in cells of the immune system provide a detailed and in-depth understanding of the effect of algal lipids on inflammatory mediators. However, the ability of algal lipids to modulate anti-inflammatory mediators or pathways that are important for the resolution of unregulated inflammation has barely been addressed and needs to be further explored.

Finally, the modulation of the inflammatory response by algal lipids in in vivo models has been explored [123,124,125,126,127], and to a lesser extent, some studies have evaluated this response in in vivo models of skin diseases [119,128,129]. Nonetheless, the few existing studies hold promise for understanding the potential of algal lipids to modulate the inflammatory response and the resulting action in skin diseases associated with chronic inflammation, as will be described. 

Regarding in vivo studies, it was demonstrated that methoxylated fatty acids (MMHDA) isolated from macroalga *Ishige okamurae* [127] and MGDG, DGDG, and SQDG fractions from microalga ETS-05 cyanobacterium [123] presented anti-inflammatory activity by reducing ear oedema (swelling) in a mouse model. The anti-inflammatory action of MMHDA has been associated with the inhibition of phospholipase A2 (PLA2), the enzyme responsible for the hydrolysis of the *sn*-2 position of membrane glycerophospholipids to liberate arachidonic acid (AA). The reduction in neutrophils was observed in the wound region of a zebrafish model when glycolipids rich in γ-linolenic acid from the microalga *Spirulina platensis* were used [124]. Extracts with omega-3 FA isolated from microalgae promoted the reduction of CD4+ T cells production of the pro-inflammatory mediators IFN-γ, TNF-α, and IL-4 and increased the secretion of IL-17A, IL-14, and TGF-β in a db/db and CD1 mouse model of diabetes Mellitus [125]. Downregulation of TNF-α was also observed, as well as decreased expression of iNOS and COX-2, when 2,4,6-trinitrobenzene sulfonic acid (TNBS)-induced colitis rats were supplemented with oxylipins extracted from *Chlamydomonas debaryana* [126].

In model studies on skin diseases, the protective effect of the ethanolic extract of *Sargassum cristaefolium* against ultraviolet-irradiated skin keratinocytes and BALB/c mice skin has been demonstrated [128]. The inhibition of ROS production and suppression of the apoptotic process in irradiated cells, such as the decrease of caspases, downregulation of COX-2, IL-1β, IL-8, IL-6, TNF-α, and INF-γ, and down-modulation of NF-κB signaling, were some of the reported mechanisms of action. *L. ochroleuca* lipid extract have been shown to reduce ear oedema, in a murine model of skin inflammation induced by the chemical sensitizer 2,4-dinitro-fluorobenzene applied to the ear of naive C57BL/6 mice [119]. A cream containing MGDG extracted from the microalga *Isochrysis galbana* has been reported to have beneficial effects in the treatment of a 12-O-tetradecanoylphorbol-13-acetate (TPA)-induced hyperplasia murine model [129]. Pre-treatment of these mice with this MGDG cream reduced skin oedema and epidermal thickness. In addition, the pro-inflammatory cytokines TNF-α, IL-1β, IL-6, and IL-17 produced in epidermal tissue were downregulated and the expression of COX-2 was inhibited. The results of this study were very promising as they involved a model of skin disease and isolated algal lipids and showed a strong anti-inflammatory effect as well as an improved skin condition. Such results highlight algal lipids as promising pharmacological strategies for the therapy of inflammatory skin pathologies. 

**Figure 1:**
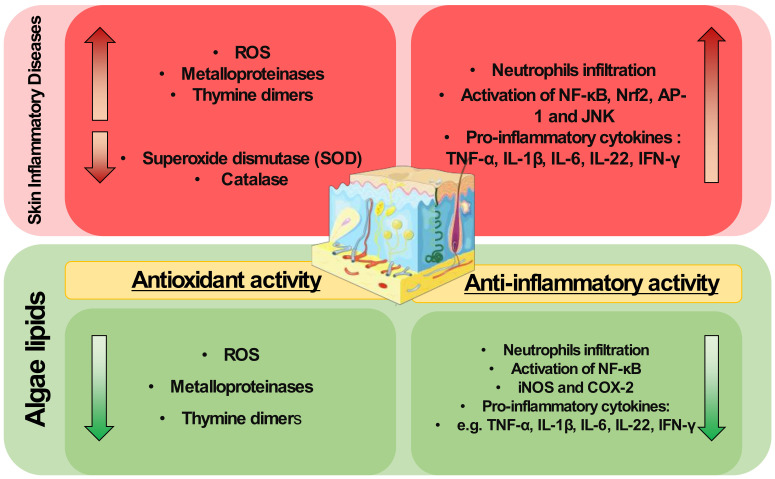
Algae lipids can target mediators responsible for the onset and progression of skin inflammatory diseases.

Algal lipids have shown anti-inflammatory potential as modulators of signaling pathways and mediators, known as the main hallmarks of inflammation, such as COX-2 and iNOS, but also the modulation of the production of cytokines (TNF-α, IL-6, IL-1β, and IFN-γ), as described in Figure 1. Indeed, as mentioned in Section 2, inflammatory skin diseases are characterized by systemic inflammation [130] with the infiltration of immune cells, such as neutrophils. Algal lipids such as glycolipids rich in γ-linolenic acid from the microalga *S. platensis* have been reported to reduce neutrophil infiltration in a wound region model of zebrafish [124]. Neutrophils are the main producers of ROS capable of activating transcription factors, such as NF-κB, responsible for the regulation of genes involved in inflammation [43]. Interestingly, the use of extracts in immune and skin cell lines reduced this pro-inflammatory pathway [110,111,123]

Studies focusing on the anti-inflammatory activity of algae extracts in models of skin disease referred above did not explore the identity of bioactive lipids. However, some of the works described above aimed to achieve the isolation of bioactive lipid species in order to find a structure–activity relationship (Table 3). To the author’s best knowledge, in many of them, the pinpointed bioactive species correspond to the most abundant species in the lipid extracts. The results collected by the analysis of the literature showed that few phospholipids and betaine lipids were bioactive. Remarkably, most research has shown glycolipids with potent bioactivity, and these bioactive lipids have been identified as carriers of omega-3 PUFA [79]. Omega-3 PUFA are precursors of lipid-derived inflammation mediators, such as endocannabinoids, useful in reducing UV damage to the skin [61]. Other important anti-inflammatory eicosanoids, such as prostaglandins, maresins, and resolvins, play an important role in attenuating the pro-inflammatory response, stimulating the anti-inflammatory activity and promoting inflammation resolution [131]. The formation and action of these lipid mediators depend largely on the prevalence of PUFA precursors, which can be provided by food, such as fish or algae, by supplementation using nutraceuticals or by topical application to enrich the skin with these healthy lipids [132,133]. Overall, the observed anti-inflammatory activity may be the result of the intrinsic biological activity of the identified glycolipids, a result of the delivery of omega-3 PUFA to produce pro-resolving mediators or a combination of the two. However, the exact mechanisms by which this anti-inflammatory activity occurs are far from being elucidated.

## 5. Concluding Remarks

Algal lipids have been shown to be effective in countering the pathophysiological processes responsible for the onset and progression of inflammatory skin diseases, such as oxidative stress and inflammation. Algal lipids have shown antioxidant activity through ROS scavenging and the modulation of important enzymes involved in the regulation of the redox state, showing the ability to attenuate the pro-oxidative state in these diseases. These extracts also contributed for the modulation of the inflammatory response at several levels, such as the modulation of signaling pathways and transcription factors such as NF-κB and MAPKs, conditioning the activation of immune cells and the production of inflammatory mediators. This modulation was demonstrated in vitro and in vivo, in different animal models of inflammatory diseases. Based on the literature collected for this review, several algal lipids are pinpointed with potential applications for skin diseases. The most studied and highlighted species were polar lipids with anti-inflammatory activity, belonging to glycolipids and phospholipids, namely MGDG, DGDG, and SQDG, and PC classes, respectively. Less reported classes included PG and DGTS species, sterols, and free and esterified FA, which also showed potential as anti-inflammatory agents. This synergistic action holds great promise for the application of algal lipids in the management of acute and chronic inflammatory skin diseases. However, the application of algal lipids in these skin diseases is an underexplored area, with few studies focusing on an integrated system, which needs to be explored in the near future.

More studies that unveil the action of lipids in models of inflammatory skin diseases (in vitro and in vivo) are needed to understand the modulating capacity of algal lipids, to fight against these diseases, such as atopic dermatitis or psoriasis. Other challenges are the lack of structural characterization of bioactive lipids, and to unveil the synergistic effect of all components of lipid extracts, as well as to reveal the structure–bioactivity relationship. These results could highlight algal lipids as therapeutic alternatives for skin diseases. The current therapeutic approaches for skin inflammatory diseases are not quite effective, are uncomfortable, and can have undesired effects, and some are expensive and not available to most patients. As reviewed in this article, algae lipids are natural antioxidant and anti-inflammatory agents that can counteract changes observed in skin inflammatory diseases, promote a return to homeostasis, and treat these conditions. They can be used as topical agents, which is preferable in the cosmeceutical industry, and are cheap to produce, extending their use to poorer countries and patients with low incomes. If these promises hold true, algal lipids could feature in the road map for the development of new pharmaceuticals and cosmeceuticals, as a treatment for skin diseases for topical or systemic administration.

## Figures and Tables

**Table 1:** Lipid extracts of algae with antioxidant activity tested *in chemico* and in vitro with potential application on skin diseases. Abbreviations: ABTS—2,2′-azino-bis(3-ethylbenzothiazoline-6-sulfonic acid, CAT—catalase, DPPH—2,2-diphenyl-1-picrylhydrazyl, FAME—fatty acyl methyl esters, HO-1—heme oxygenase-1, IL-6—interleukin-6, MAPK—mitogen-activated protein kinase, MMP—metalloproteinase, NHFD—normal human dermal fibroblasts, ROS—reactive oxygen species, SOD—superoxide dismutase, TGF-1—tumor growth factor-1, UVB—ultraviolet B.

Studies	Mechanism	Assay	Identified Lipids	Algae Species	Ref.
*in chemico*	Free radical scavenging	ABTS, DPPH, hydroxyl radical, superoxide anion	Polar lipids, neutral lipids, FAME	Macroalgae: *Bifurcaria bifurcata, Codium tomentosum, Fucus vesiculosus, Gracilaria gracilis Grateloupia turuturu, Palmaria palmata, Porphyra dioica Sargassum muticum, Solieria chordalis, Ulva rigida* Microalgae: *Chlorella vulgaris, Chlorococcum amblystomatis, Nannochloropsis oceanica, Phaeodactylum tricornutum, Scenedesmus intermedius Scenedesmus obliquus, Spirulina* sp., *Tetraselmis chui*	[69,70,82,83]
in vitro	Detoxify intracellular ROS	Increased the expression of Nrf2 in irradiated HaCat cells Upregulate target antioxidant enzymes Cu/Zn SOD, CAT, and HO-1	Crude ethanolic extract	Macroalga: *Carpomitra costata*	[84]
	Free radical scavenging	Superoxide generation on peritoneal leukocytes	Sulfoquinovosylacylglycerols	Microalgae: *Porphyridium cruentum*	[86]
	Inhibition of ROS	Photoprotective against UVB in NHDF	Crude ethyl acetate extract	Microalga: *Ettlia* sp. YC001	[85]
	Enzyme/protein expression	Downregulation of expression of MMPs	Crude ethanolic extract	Microalga: *Arthrospira platensis*	[87]
	Enzyme/protein expression	Downregulation of expression of MMPs, IL-6 and TGF-1 in human dermal fibroblast Modulate MAPK in irradiated HaCat cells	Fucosterol	Macroalga: *Sargassum fusiforme*	[88,89]

**Table 2:** Lipid extracts of algae with anti-inflammatory activity tested *in chemico,* in vitro and in vivo with a potential application on skin diseases. Abbreviation: COX-2—cyclooxygenase-2, DGDG—digalactosyldiacylglycerol, DGLA—dihomo-γ-linolenic acid, DGTS—diacylglyceryltrimethylhomoserine, DNFB—2,4-dinitrofluorobenzene, IL-6—interleukin-6, MGDG—monogalactosyldiacylglycerol, MGMG—monogalactosylmonoacylglycerol, MGTS—monoacylglyceryltrimethylhomoserine, MMHDA—methoxylated fatty acids, NF-κB—nuclear factor kappa-light-chain-enhancer of activated B cells, NO—nitric oxide, PBMC—peripheral blood mononuclear cell, PC- phosphatidylcholine, PG—phosphatidylglycerol, PGE2—prostaglandin E2, PLA2—phospholipase A2, SQDG—sulfoquinovosyldiacylglycerol, TLR—Toll-like receptor, TNBS—2,4,6-trinitrobenzene sulfonic acid, TNF-α—tumor necrosis factor-α, TPA—12-O-tetradecanoylphorbol-13-acetate.

Studies	Action	Model	Identified Lipids	Algae Species	Ref.
*In chemico*	COX-2 inhibition	COX-2 kit assay	Polar lipids	Macroalgae: *Codium tomentosum, Fucus vesiculosus Gracilaria gracilis, Palmaria palmata, Porphyra dioica, Ulva rigida, * Microalgae: *Chlorella vulgaris, Chlorococcum amblystomatis, Gloeothece sp., Skeletonema* sp., *Tetraselmis sp. mutants*	[69,92,93,94,95]
In vitro	NO inhibition	Raw 264.7	Polar and non-polar lipids; PC, PG, DGDG, DGTS, MGDG, MGMG, SQDG classes; Free and ethyl esterified DGLA	Macroalgae: *Chondrus crispus, Lobophora sp.Palmaria palmata*, Microalgae: *Chlorella sorokiniana Lobosphaera incisa, Nannochloropsis granulata, Tetraselmis chui,*	[96,97,98,99,100,101,102,103]
	Decrease in PGE2 Downregulation of COX-2	Raw 264.7; White blood cells; Epidermal cells	Crude ethanolic extracts; lipid extracts rich in PC; free and ethyl esterified DGLA	Macroalgae: *Laminaria ochroleuca* Microalgae: *Chlorella vulgaris, Chloromonas reticulata, Lobosphaera incisa Micractinium* sp., *Phaeodactylum tricornutum,*	[101,104,105,106,107,119]
	Downregulation of mRNA expression of pro-inflammatory cytokines Downregulation of cytokines levels: TNF-α, IL-6, IL-1α, and IL-1β	THP-1; PBMC; Epidermal cells; HaCaT cells	Crude ethanolic extracts; lipid extracts; lipid extracts rich in MGDG, DGDG and SQDG; Lipid extracts rich in PC; LPC(16:0); oxylipins; ergosterol and 7-dehydroporiferasterol; free and ethyl esterified DGLA	Macroalgae: *Chondrus crispus, Laminaria ochroleuca, Palmaria palmata, Porphyra dioica, Prasiola japonica * Microalgae: *Aurantiochytrium mangrovei, Chlamydomonas debaryana, Chlorella vulgaris, Chloromonas reticulata, Cylindrotheca closterium, Dunaliella tertiolecta, Micratinium* sp., *Nannochloropsis gaditana, Nitzschia palea, Phaeodactylum tricornutum, Lobosphaera incisa Spirulina maxima, Pavlova lutheri, Tetraselmis suecica,*	[84,101,104,105,106,107,110,111,112,113,114,115,116,118,119,121,122]
	Inhibition of pro-inflammatory signaling pathways mediated by TLR and NF-κB	THP-1	Lipid extracts rich in MGDG, DGDG, and SQDG	Macroalgae: *Chondrus crispus, Palmaria palmata, Porphyra dioica* Microalgae: *Pavlova lutheri*	[110]
In vivo	Attenuation of ear oedema	PLA2 kit assay; Mice with ear oedema; DNFB-induced in naive C57BL/6 mice	MMHDA; Lipid extracts rich in PC; MGDG, DGDG, and SQDG fractions	Macroalgae: *Ishige okamurae*, *Laminaria ochroleuca* Microalgae: ETS-05 cyanobacterium.	[119,123,127]
	Neutrophil gathering in the wound region	Wounded zebrafish model	Glycolipids rich in γ-linolenic acid	Microlagae: *Spirulina platensis*	[124]
	Inhibition of pro-inflammatory cytokines production: TNF-α, IL-6, IL-8, IFN- γ, IL-1β, IL-17	db/db and CD1 mice model of diabetes mellitus; TNBS-induced colitis rats; BALB/c mice skin; TPA-induced hyperplasia murine model	Crude ethanolic extract; omega-3 fatty acids; oxylipins; MGDG cream	Macroalgae: *Sargassum cristaefolium* Microalgae: *Chlamydomonas debaryana, Isochrysis galbana*	[125,126,128,129]
	Downregulation of iNOS and COX-2, and decrease in NO and PGE2 production	TNBS-induced colitis rat; BALB/c mice skin; TPA-induced hyperplasia murine model	Crude ethanolic extract; oxylipins; MGDG cream	Macroalgae: *Sargassum cristaefolium* Microalgae: *Chlamydomonas debaryana, Isochrysis galbana*	[126,128,129]

**Table 3:** Polar lipid species from microalgae and macroalgae with reported anti-inflammatory activity. Abbreviations: DGTS—diacylglyceryltrimethylhomoserine, MGTS—monoacylglyceryltrimethylhomoserine, MGDG—monogalactosyldiacylglycerol, MGMG—monogalactosylmonoacylglycerol, DGDG—digalactosyldiacylglycerol, SQDG—sulfoquinovosyldiacylglycerol, PC—phosphatidylcholine, LPC—lysophosphatidylcholine, PG—phosphatidylglicerol, C—total number of carbon atoms on fatty acyl chains, N—total number of double bonds on the fatty acyl chains.

Lipid Class	Lipid Species (C:N)	Molecular Species (*sn*-1/*sn-*2)	Algae Species	Reference
Betaine lipids	DGTS (34:5)	DGTS (20:5/14:0)	*Nannochloropsis granulata*	[102]
	DGTS (36:5)	DGTS (20:5/16:0)		
	DGTS (36:6)	DGTS (20:5/16:1)		
	DGTS (38:7)	DGTS (20:5/18:2)		
	DGTS (40:9)	DGTS (20:5/20:4)		
	DGTS (40:10)	DGTS (20:5/20:5)		
	MGTS (20:5)	MGTS (20:5)	*Nannochloropsis* sp.	[134]
Glycolipids	MGDG (34:3)	MGDG (16:0/18:3)	ETS-05 cyanobacterium	[123]
	MGDG (34:4)	MGDG (18:4/16:0)	*Chondrus crispus*	[97]
	MGDG (34:5)	MGDG (20:5/14:0)	*Nannochloropsis granulata*	[100]
	MGDG (34:7)	MGDG (18:3/16:4)	*Tetraselmis chui*	[99]
	MGDG (34:8)	MGDG (18:4/16:4)		
	MGDG (36:4)	MGDG (20:4/16:0)	*Chondrus crispus*	[97]
	MGDG (36:5)	MGDG (20:5/16:0)	*Chondrus crispus*, *Nannochloropsis granulata*	[97,100]
	MGDG (36:6)	MGDG (20:5/16:1)	*Nannochloropsis granulata*	[100]
	MGDG (38:7)	MGDG (20:5/18:2)	*Porphyridium cruentum*	[86]
	MGDG (40:8)	MGDG (20:4/20:4)	*Chondrus crispus*	[97]
	MGDG (40:9)	MGDG (20:5/20:4)	*Chondrus crispus*, *Porphyridium cruentum*	[86,97]
	MGDG (40:10)	MGDG (20:5/20:5)	*Chondrus crispus*, *Nannochloropsis granulata*	[97,100]
	MGMG (16:2)	MGMG (16:2)	*Chlorella sorokiniana*	[103]
	MGMG (16:3)	MGMG (16:3)		
	DGDG (34:4)	DGDG (16:0/18:4)	ETS-05 cyanobacterium	[123]
	DGDG (34:5)	DGDG (20:5/14:0)	*Nannochloropsis granulata*	[100]
	DGDG (36:4)	DGDG (20:4/16:0)	*Chondrus crispus*, *Porphyridium cruentum*	[86,97]
	DGDG (36:5)	DGDG (20:5/16:0)	*Chondrus crispus*, *Nannochloropsis granulata*	[97,100]
	DGDG (36:6)	DGDG (20:5/16:1)	*Nannochloropsis granulata*	[100]
	DGDG (38:7)	DGDG (20:5/18:2)	*Porphyridium cruentum*	[86]
	DGDG (40:10)	DGDG (20:5/20:5)	*Nannochloropsis granulata*	[100]
	DGDG (40:9)	DGDG (20:5/20:4)	*Porphyridium cruentum*	[86]
	SQDG (34:3)	SQDG (18:3/16:0)	ETS-05 cyanobacterium	[123]
	SQDG (34:5)	SQDG (20:5/14:0)	*Palmaria palmata*	[98]
	SQDG (36:5)	SQDG (20:5/16:0)		
Phospholipids	PC (40:10)	PC (20:5/20:5)	*Palmaria palmata*	[98]
	LPC (16:0)	LPC (16:0)	*Cylindrotheca closterium*	[118]
	PG (34:2)	PG (16:0/18:2)	ETS-05 cyanobacterium	[123]
	PG (36:6)	PG (20:5/*trans*-16:1)	*Palmaria palmata*	[98]
		PG (20:5/16:1)		
